# Intramedullary nailing for pertrochanteric fractures of proximal femur: a consecutive series of 323 patients treated with two devices

**DOI:** 10.1186/s13018-019-1506-1

**Published:** 2019-12-18

**Authors:** Pompeo Catania, Daniele Passaretti, Giorgio Montemurro, Simone Ripanti, Stefano Carbone, Vittorio Candela, Michele Carnovale, Stefano Gumina, Francecsco Pallotta

**Affiliations:** 1 Department of Orthopaedics and Traumatology, San Giovanni-Addolorata Hospital, Rome, Italy; 2 Department of Orthopaedics and Traumatology, C.T.O. Hospital, Rome, Italy; 3 Department of Orthopaedics and Traumatology, Clinica San Feliciano, Rome, Italy; 4 Department of Orthopaedics and Traumatology, Sapienza University of Rome, ICOT, Latina, Italy

**Keywords:** Pertrochanteric fractures, Femur intramedullary nailing, Elderly fracture treatment, Gamma nail, Elos nail

## Abstract

**Introduction:**

Pertrochanteric fractures (PFs) frequently affect the lower limb of osteoporotic patients and represent an important cause of morbidity and mortality in the elderly. In this prospective randomized controlled trial, we have compared functional and radiological results and complications of patients affected by PFs treated with two intramedullary proximal femoral nails.

**Materials:**

We enrolled 323 subjects with PFs, classified according to AO/OTA system as 31.A1 (pertrochanteric simple) and 31.A2 (pertrochanteric multifragmentary). Patients were divided into two groups according to the osteosynthesis devices: group A, Elos-Intrauma® nail (155 cases) and group B, Gamma 3-Stryker® nail (168 cases). Pre-operatively, the baseline characteristics of each patient (gender, age, weight and BMI) were collected. Intraoperative blood loss, subjective pain by visual analogue scale (VAS), esthetic satisfaction, functional scores of the hip by Harris Hip Score (HHS), and Western Ontario and McMaster Universities Arthritis Index (WOMAC) were noted. The post-operative degree of fracture reduction was assessed. Each patient had a minimum follow-up of 12 months.

**Results:**

The study group was composed of 106 male and 217 female with an average age of 85.4 (range, 65–90, standard deviation (SD) 5.95) years. No statistical differences about sex and age distribution were noted between the two groups. Group A reported lower intraoperative blood loss, 45 ml vs 51 ml, respectively (*p* < 0.001). There was not any statistical difference about operative time. Group A had a better reduction of fracture (*p* = 0.0347). The greatest difference was detectable comparing subgroups 31.A2 (*p* = 0.032). There were no statistical differences about complication frequency and the overall rate was 25% (80 cases). Finally, there was no difference in terms of VAS, HHS, and WOMAC score between the two groups on each follow-up. Patients of group A showed a higher subjective satisfaction index at 1 post-operative year, 7.42 (SD 1.19) vs 6.45 (SD 1.35) of group B (*p* < 0.001).

**Conclusion:**

Elos® nail is a reliable device on a short-term follow-up and represents an alternative choice to the Gamma 3® nail, a well-known and appreciated system for over 25 years.

## Introduction

Pertrochanteric fractures (PFs) are frequent lesions of the lower limb, generally associated with low energy trauma of the elderly patient, older than 65 years, with osteoporosis [1, 2], and they represent an important cause of morbidity and mortality in these subjects [3, 4].

PFs are surgically treated with a closed reduction and internal fixation (CRIF) osteosynthesis by a cervical-cephalic nail or a sliding hip screw [5–13]. Intramedullary nail is the preferred synthesis because it allows a reduction of the time of surgery, a stable synthesis and an early patient mobilization [14]. In particular, these considerations are valid for more stable PFs, classified with AO/OTA [15] pattern 31-A1 (pertrochanteric simple) and 31-A2 (pertrochanteric multifragmentary).

Since PFs are an important health and economic problem, the use of constantly evolving effective tools that allow us to reduce patient hospitalization and public spending is essential.

Therefore, we carried out a prospective randomized controlled trial in which we compared both functional and radiological outcomes together with the complication rates of patients treated with two different intramedullary proximal femoral nails.

## Materials and methods

From July 1, 2015, to October 31, 2017, 741 consecutive patients with pertrochanteric fracture were enrolled. All patients were treated in the Orthopedics and Traumatology department of S. Giovanni-Addolorata Hospital and ICOT Hospital.

Inclusion criteria were as follows: age between 65 and 90 years old; fracture pattern AO/OTA [15] 31.A1 (pertrochanteric simple) or 31.A2 (pertrochanteric multifragmentary). Fracture classification was performed by authors, experienced orthopedics (CP, MG, GS), on the basis of AP and axial x-ray of proximal femur; surgery performed within 48 h from the trauma; a minimum post-operative follow-up of 12 months; a good cognitive and motor abilities, being able to walk without aids before the femur fracture.

Exclusion criteria were as follows: pathological fractures or the presence of an on-going metastatic disease; polytraumatized subjects; the presence of a high grade of hip osteoarthrosis; hematological disease or therapy with anticoagulants and antiplatelet drugs; patients with American Society of Anaesthesiologists score (ASA score) of 5.

We therefore excluded 418 subjects who did not meet all inclusion criteria: 317 were treated with a different device; 29 had a 31.A3 pattern [15] (intertrochanteric fracture), which was treated with a long intramedullary nail or with a sliding hip screw; finally, 72 patients presented one or more exclusion criteria.

Participants were divided by randomization into two groups, A and B, according to their close reduction internal fixation (CRIF) by Elos-Intrauma® nail or Gamma 3-Stryker® nail, respectively. Randomization was done by a clinician not enrolled in the study using random blocks.

Pre-operatively, the baseline characteristics of each subject (gender, age, weight, and BMI) were collected, and the fracture pattern was classified according to the AO/OTA system [15].

Surgery was performed by three of the authors (CP, GM, and GS). Cefazolin 2 g was administered 30 min before surgery. Patients received a spinal anesthesia and were operated in a supine position with the use of a traction table. The pre- and intraoperative anatomical reduction of the fracture was controlled by fluoroscopy. The surgical technique was in accordance with the protocol described by Mereddy [16] and Zhong [17]. The nail was distally stabilized with static or dynamic screw according to fracture. For patients of group A, with a 31.A2 fracture, in order to increase the stability of the synthesis, an antirotational cervico-cephalic screw was applied, in addition to the cephalic screw. The duration of surgery (time from the cutaneous incision to the surgical suture) was recorded. In patients with narrowing of proximal femur medullary canal, obstructing the nail entry, a bore with burr was performed to obtain a medullary diameter 1.5–2 mm greater than that of the nail.

Intraoperative blood loss was recorded. The amount of losses was calculated as “Blood Loss = [(Used gauzes weight − Clean gauzes weight) − weight of Physiological Solution used as washing]”. We did not use other swab systems, such as aspiration with cannula, to make this measurement as much reliable as possible.

Subjects underwent a standard post-operative rehabilitative protocol (when allowed by the patient’s clinical status and osteosynthesis stability). It consisted of 1 day of bed rest with passive mobilization of the lower limb, followed by an assisted walking with tolerance weight bearing. Usually, patients carried out rehabilitation protocol for the affected hip for 1 month post-operatively.

Regarding post-operative clinical evaluations, follow-ups were performed every 5 days for the first 15 days (to check the general conditions and surgical suture), at 1, 6, and 12 months. At each evaluation, we noted the following: subjective pain by visual analogue scale (VAS); esthetic satisfaction by a subjective index, which consisted in a score value included between 0 and 10, where 0 and 10 corresponded to the minimum and maximum, respectively; functional scores of the hip by two well-known systems, the Harris Hip Score (HHS) [18]; and the Western Ontario and McMaster Universities Arthritis Index (WOMAC) [19].

Regarding radiographic evaluations, an x-ray control was performed at the immediate post-operative and at follow-up at 1, 3, 6, and 12 months. We assessed the degree of fracture reduction through post-operative view, according to Baumgaertner classification, modified by Fogagnolo [20]. The consolidation delay was classified as *Mal-union* if there was less than 50% bone contact between proximal and distal fragment at the x-ray or a collapse of the cervico-diaphyseal angle, < 120°; *Non-union* if there was a total lack of bone union after 6 months post-operatively.

### Statistical analysis

Calculation of sample size was done using G*Power 3.1.9.3 software (Heinrich-Heine-University, Dusseldorf, Germany). According to post hoc Wilcoxon–Mann–Whitney test, assuming an α-value of 0.05 (sensitivity of 95%) and a sample size group of 165 patients and 168 patients, the power achieved is 94% (*β*-value = 0.06). Shapiro–Wilk test was used to verify normal distribution of samples. For unpaired group comparisons, the Mann–Whitney *U* test was used. All statistical tests were two-sided. *p* values < 0.05 were considered as statistically significant. All metric variables are reported as mean and standard deviation. All statistical calculations were performed using R 3.5.1 software (R Foundation for Statistical Computing, Vienna, Austria, https://www.r-project.org).

The *x*^2^ test was applied to verify a significant statistical difference in terms of complications and fracture reduction. The significance level was set at *p* < 0.05.

## Results

The final number of subjects enrolled in our study was 323 (155 in group A and 168 in group B). Between groups A and B, no statistical differences emerged about sex and age distribution. Seventeen percent of subjects (55 cases) had a type 31.A1 fracture (28 in group A and 27 in group B), and 83% (268 cases) had a 31.A2 fracture (127 in A and 141 in B). All data are reported in Table 1.

**Table 1:** Baseline characteristics of the study sample

	*n* (%)	Range (mean ± SD)
Group A	155	
Age		65–90 (85.1 SD 6.42) ys
Gender	M 51–F 104	
31.A1	28 (18.1%)	
31.A2	127 (81.9%)	
Group B	168	
Age		66–90 (85.7 SD 5.48) ys
Gender	M 55–F 113	
31.A1	27 (16.1%)	
31.A2	141 (83.9%)	

*n* number; *SD* standard deviation; *ys* years

Groups A and B had a mean intraoperative blood loss of 45 ml (± 7.92 SD) and 51.3 ml (± 10.54 SD), respectively. A significant difference was found (*p* < 0.001). Statistical differences were also found according to the boring of femur canal, performed in 3 and 24 subjects of groups A and B, respectively (*p* < 0.001). Regarding surgery duration, in group A, the mean time was 43 min (± 7.19 SD), and in Group B was 42 (± 7.4 SD), (*p* = 0.63). About post-operative fracture reduction, in group A emerged a statistical better result, (*p* = 0.035). The greatest difference was detectable comparing subgroups 31.A2 (multifragmentary of great trochanter and supero-lateral metaphyseal cortex), with a *p* value of 0.032. No difference was found comparing 31.A1 subgroup in two groups, *p* = 0.92. Data regarding fracture reduction is shown in Table 2.

**Table 2:** Post-operative fracture anatomical reduction according to Fogagnolo classification

Reduction	Group A n. (31.A1 + 31.A2)	Group B n. (31.A1 + 31.A2)	*p* value
Good	108	94	*0.0347
Acceptable	38	57	
Poor	9	17	
Reduction	Group A n. (31.A1)	Group B n. (31.A1)	*p* value
Good	20	18	0.92
Acceptable	7	8	
Poor	1	1	
Reduction	Group A n. (31.A2)	Group B n. (31.A2)	*p* value
Good	88	76	*0.032
Acceptable	31	49	
Poor	8	16	

*n* number of patient; *statistically significant *p* value

There were no statistical differences about complications rate. Overall rate was 24.8% (80 cases, 37 in group A and 43 in group B). We observed 4 lag screw cut-outs in group A and 8 in group B; 5 and 2 local infections were registered in groups A and B, respectively; 2 consolidation delays in group A and 4 in group B (Malunion with cervical-metaphyseal varus scomposition, < 120°); 1 proximal screw dislocation in the soft tissue in group B; 1 femoral fracture close to the nail distal tip in group A; 2 nail breakage in group A. Due to these adverse events, 8 and 11 patients of group A and B, respectively, were submitted to re-surgery. Finally, 23 subjects in group A and 28 in group B died for other reasons before the first post-operative (po) year.

As shown in Table 3, no statistical difference was reported comparing VAS score at 1 day (*p* = 0.074), 15 days (*p* = 0.098), 1, 6, and 12 months post-operatively (*p* = 0.1; 0.109; 0.421, respectively), HHS at 6 and 12 months post-operatively (*p* = 0.438; 0.56, respectively); WOMAC at 6 and 12 months post-operatively (*p* = 0.399; 0.777, respectively) between the two groups. Finally, a significant difference was found in the subjective satisfaction index. Patients of group A reported a higher index compared with group B, with mean values of 7.42 (SD 1.19) and 6.45 (SD 1.35), (*p* value < 0.001).

**Table 3:** Subject pain and functional scores of the hip at each post-operative follow-up

		Mean (SD) Group A	mean (SD) Group A	*p* value
VAS	1 d	5.95 (1.43)	6.18 (1.42)	0.074
	15 d	4.33 (1.51)	4.57 (1.63)	0.098
	1 m	3.3 (0.46)	3.38 (0.82)	0.1
	6 m	0.74 (0.56)	0.63 (0.55)	0.109
	12 m	0.48 (0.5)	0.43 (0.5)	0.421
HHS	6 m	85.14 (7.55)	84.25 (8.19)	0.438
	12 m	89.34 (7.56)	89.84 (7.55)	0.56
WOMAC	6 m	79.2 (7.88)	78.31 (8.39)	0.399
	12 m	84.61 (7.69)	84.82 (7.75)	0.777

*VAS* visual analogue, *HHS* Harris Hip Score, *WOMAC* Western Ontario and McMaster Universities Arthritis Index, *d* post-operative day/days, *m* post-operative month/months, *SD* standard deviation

## Discussion

PFs represent the most frequent cause of hospitalization in an orthopedic department [11]. The treatment of these fractures must be aimed to early assisted walking restoration, to reduction of hospitalization time and of morbidity of the patient. Factors associated with a better outcome are an early treatment, an anatomical reduction of fragments, a stable osteosynthesis, an early mobilization, and a full weight bearing and walking [21, 22]. Following these principles, we carried out a randomized prospective study and we compared functional and radiological outcomes and complication rate in patients treated with two different proximal femoral nail for a pertrochanteric fracture.

**Fig. 1:**
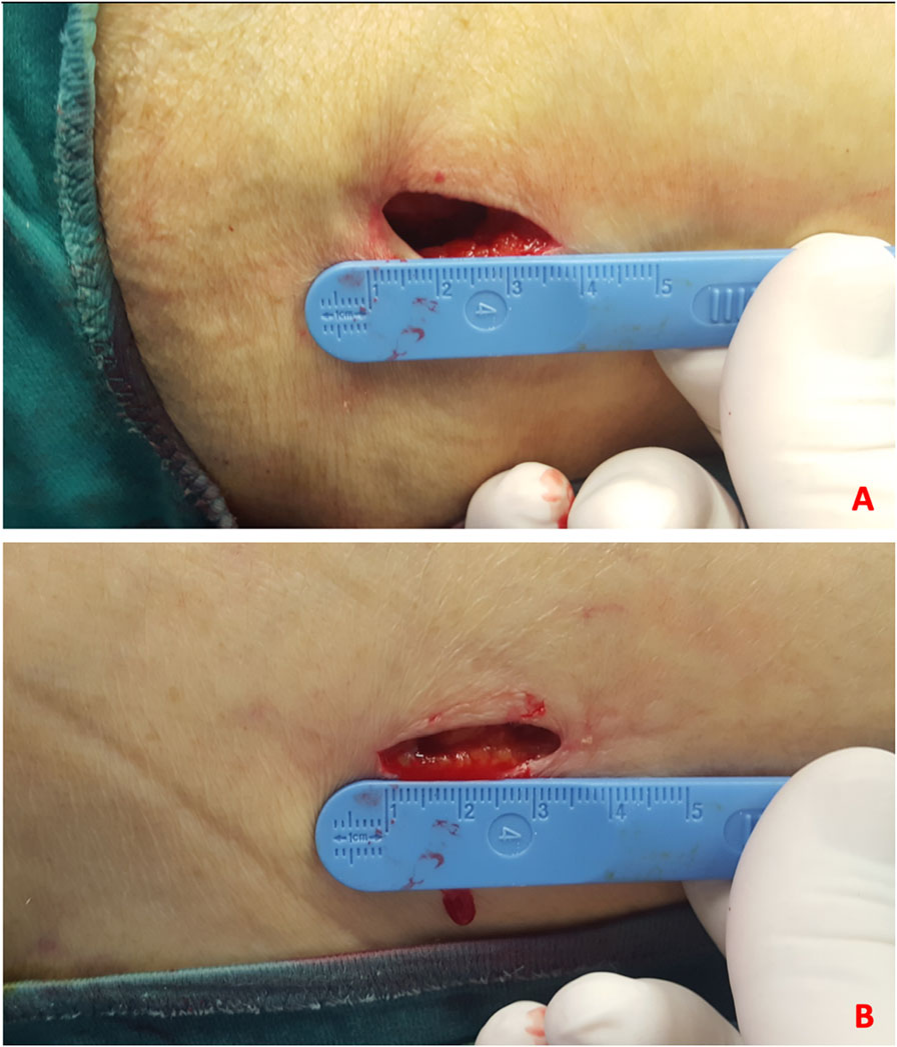
Elos® nail can often require only 2 mini-open surgical accesses. **a** Proximal access, **b** distal access

**Fig. 2:**
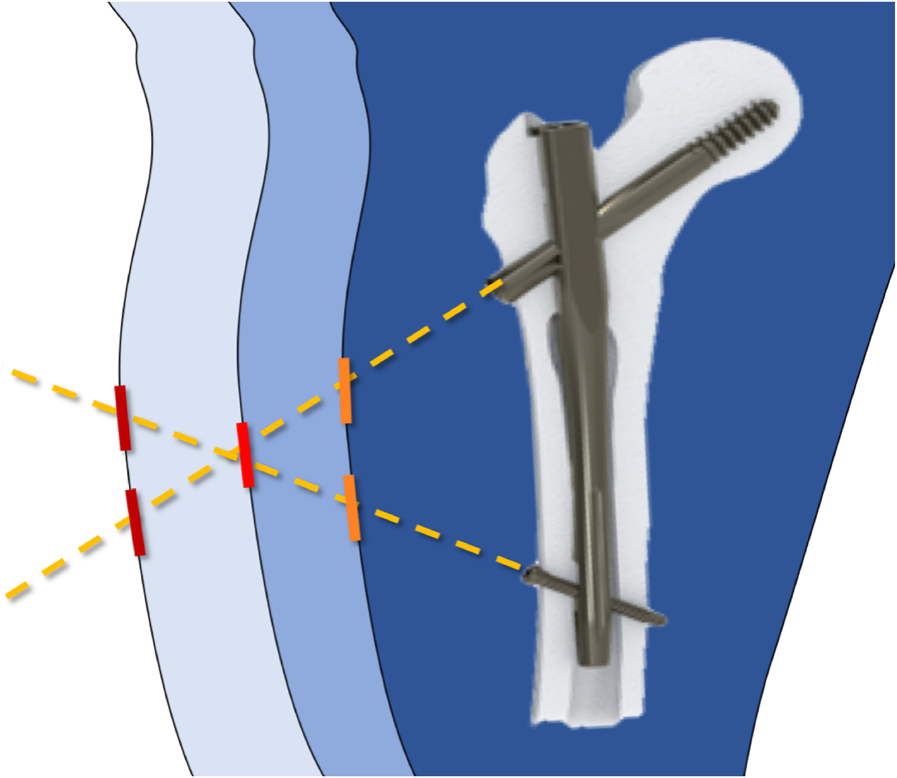
The cephalic and the distal locking screws of the Elos® nail can often be inserted through a single cutaneous access, according to the anthropometric characteristics of the patient, as the thigh circumference

**Fig. 3:**
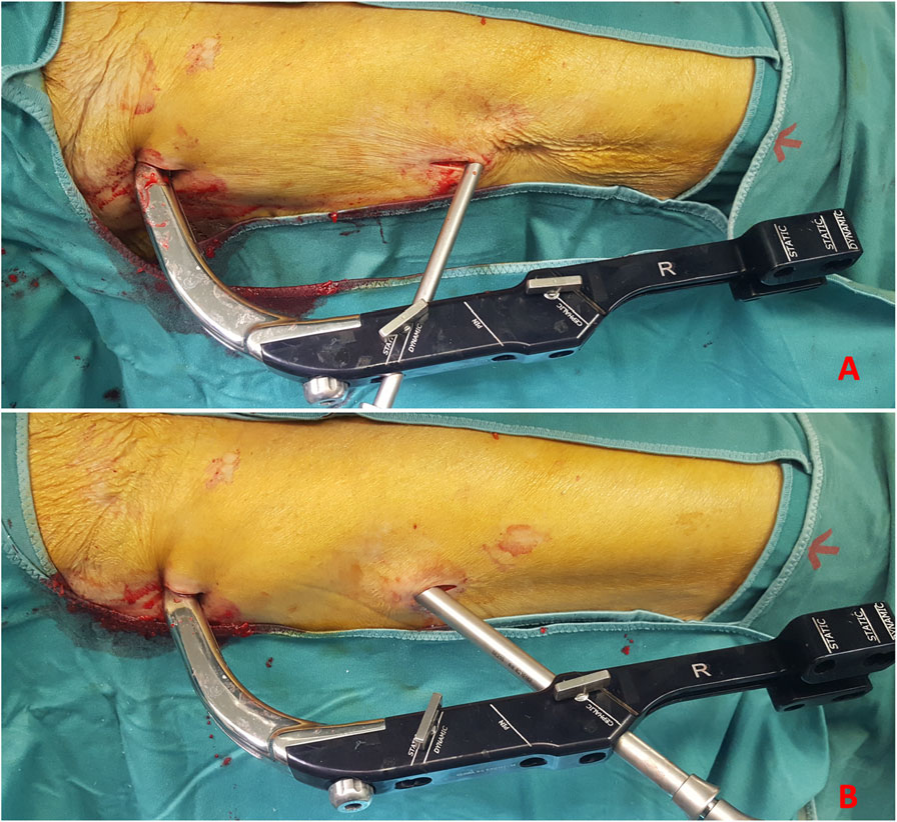
**a** Cephalic screw insertion; **b** distal locking screws insertion

Considering the baseline demographic characteristics, we found no significant differences between the two groups. This result is due to the randomization process of the patients. A lower intraoperative blood loss was detected in patients of group A, even if the real difference was minimal (< 10 ml). This result may be due to the design of the Elos® nail, in which the cephalic screw and the distal locking screw (both in the static and dynamic position) can be inserted through a single, minimally invasive, cutaneous access permitting this nail to be implanted with only 2 mini-open surgical accesses (Fig. 1a, b). However, in case of obese patients, as shown in Figs. 2 and 3a, b two cutaneous accesses will be needed to introduce both cephalic and distal screws. In our sample, two different accesses were needed in only 25 cases. In addition, due to the smaller diameter compared with Gamma 3® (10 mm vs 11 mm), Elos® nail required fewer bores of the femoral canal (3 vs 24); it could justify also the lower intraoperative blood loss.

**Fig. 4:**
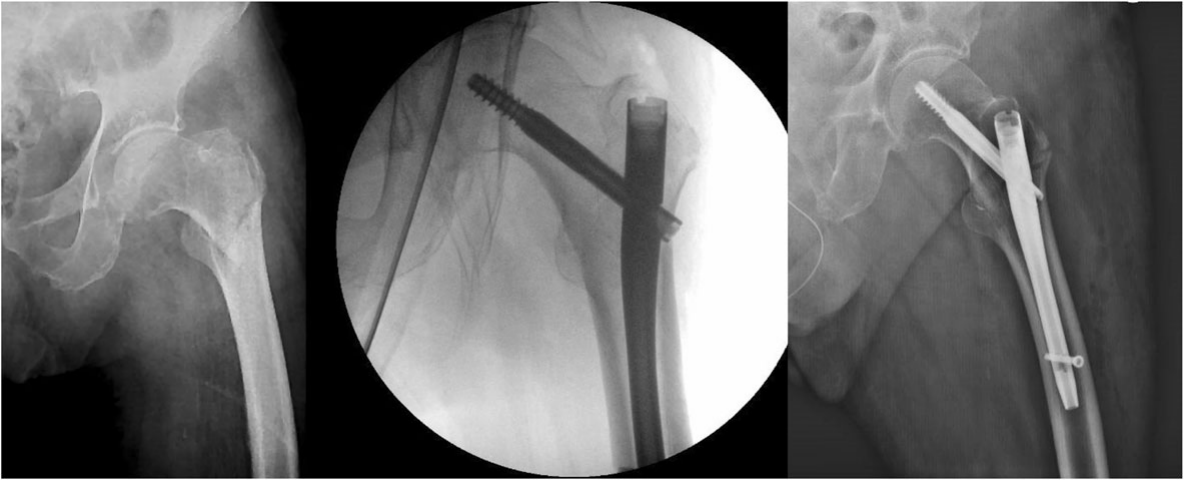
Pre-operative and post-operative x-rays of a patient with a pertrochanteric fracture (31.A1, pertrochanteric simple) treated with Gamma 3® nail

**Fig. 5:**
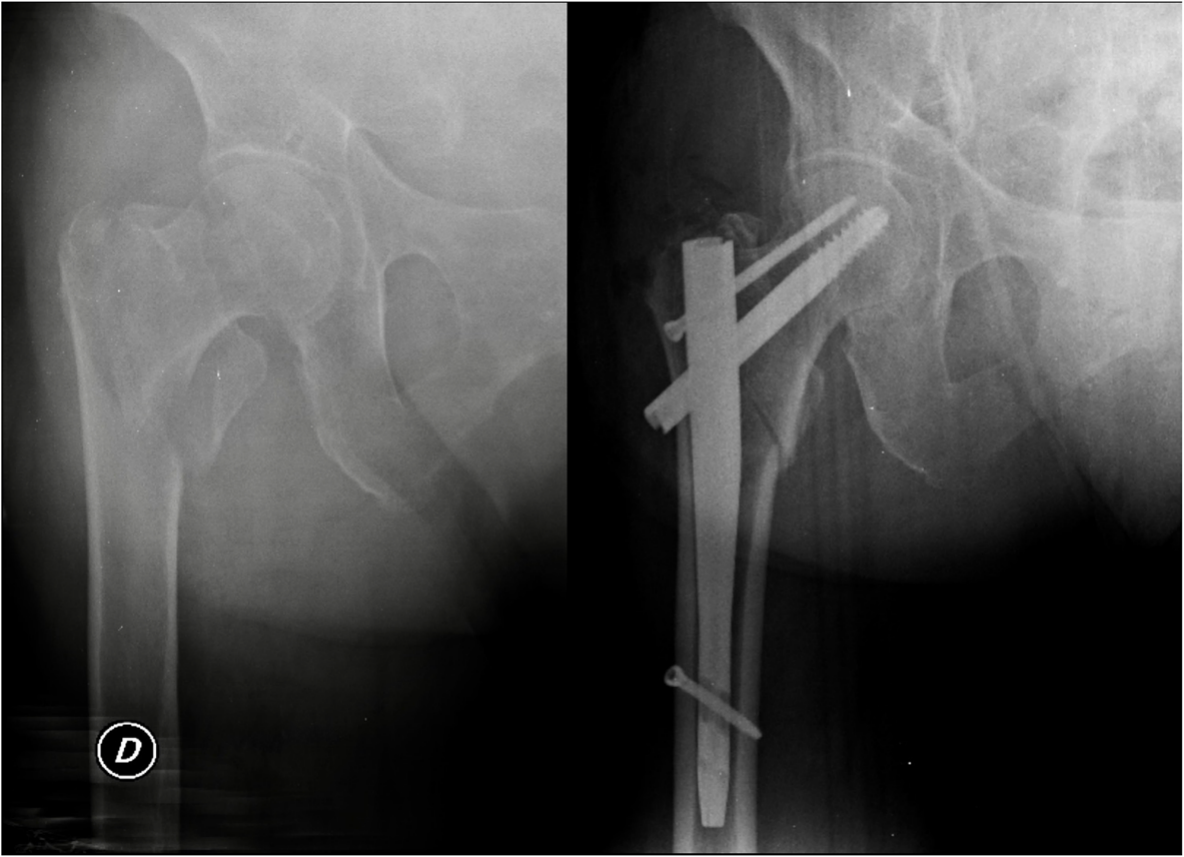
Pre-operative and post-operative x-rays of a patient with a pertrochanteric fracture (31.A2, pertrochanteric multifragmentary) treated with Elos® nail

Duration of surgery was similar in the two groups, the two devices were applied following the same technique [16, 17] and surgery was performed by the same surgeons. Regarding fracture reduction, the result obtained in group A was better than that in group B. This data could be due to the different nail interfragmentary compression systems of the cervical-metaphyseal fracture. Gamma 3® nail reduces the fracture backing the cephalic screw; the Elos® nail, on the other hand, reduces the fracture by medially translating the whole introducer handpiece-nail system. This feature is particularly advantageous in patients with multifragmention of greater trochanter and supero-lateral metaphyseal cortex (pattern 31.A2 according to the AO classification [ 15]). Figures 4 and 5 show pre and post-operative x-rays of patients treated with Gamma 3® and Elos® nails, respectively.

The overall complication rate in our study was 25%; it is similar to those of other authors [11, 23–26]. According to the literature, the cut-out of the lag screw is the most frequent complication of the nail for proximal femur fracture [11, 23, 27–31] with an occurrence rate between 1 and 5% of surgeries [11, 23–26] causing the 84% of failures and revision surgery of these devices [32]. We found this complication in 3.7% of patients and only 4 of them occurred in group A. This finding may be due to the optional antirotational screw of the Elos® nail in addition to the cephalic screw. In the literature, it is reported that the three main factors causing the screw cut-outs are the presence of a multifragmentary and unstable fracture, the non-anatomical reduction of the fragments and of the cervico-diaphyseal angle, and the wrong position of the lag screw [29]. A combination of all three factors leads to a high probability of cut-out [23]. For a good reduction, the cervico-diaphyseal angle must be > 130°; the cephalic screw must be positioned in the inferior-central quadrant or, alternatively, in the inferior-anterior or inferior-posterior quadrants; finally, the distance between medial cortex of the femoral head and the screw’s apex must never be greater than 2.5 cm [23].

Regarding the perceived pain and the functional outcomes assessed by HHS and WOMAC scores, the two groups did not show differences in both 6- and 12-month post-operative evaluation. Probably, this result was obtained because all patients were subjected to the same rehabilitation protocol, and because the two nails have an overlapping reliability.

Finally, the subjective esthetic result was better in group A. Patients treated with Elos® nail reported a greater liking at follow-up at 1 year, probably because they had, in most cases, only two surgical incisions, compared with three incisions of the group B patients.

Our study has some limitations; the low number of enrolled patients and the duration of the minimum follow-up at only 1 year, which does not allow the long-term evaluation of outcomes and complications, are the main ones.

## Conclusion

In this study, we compared functional and radiological results and complications of patients who underwent to osteosynthesis by Elos® or Gamma 3® nails, for a pertrochanteric fracture. In the Elos-group, we have registered a lower intraoperative blood loss, a better anatomical reduction of the fracture, particularly in patients with multifragmention of great trochanter and supero-lateral metaphyseal cortex, and a greater esthetic result. On the other hand, complication rate, post-operative perceived pain, and functional outcomes were similar between the two groups.

According to our short-term follow-up results, we propose the Elos® nail as a reliable device. Overall, it may represent a valid choice for the orthopedic surgeon.

## Data Availability

Data sharing is not applicable to this article. Please contact author for data requests.

## Abbreviations

ASA score: American Society of Anaesthesiologists score
CRIF: Closed reduction and internal fixation
HHS: Harris Hip Score
PF: Pertrochanteric fractures
po: Post-operative
SD: Standard deviation
VAS: Visual analogue scale
WOMAC: Western Ontario and McMaster Universities Arthritis Index

## References

[1] Bjorgul K, Reikeras O (2007). Incidence of hip fracture in southeastern Norway: a study of 1,730 cervical and trochanteric fractures. Int Orthop.

[2] Finsen V, Johnsen LG, Trano G, Hansen B, Sneve KS (2004). Hip fracture incidence in central Norway: a followup study. Clin Orthop Relat Res.

[3] Braithwaite RS, Col NF, Wong JB (2003). Estimating hip fracture morbidity, mortality and costs. J Am Geriatric Soc.

[4] Weller I, Wai EK, Jaglal S (2005). The effect of hospital type and surgical delay on mortality after surgery for hip fracture. JBJS Br.

[5] Parker MJ, Handoll HH (2002). Gamma and other cephalo-condylic intramedullary nails versus extramedullary implants for extracapsular hip fractures. Cochrane Database Syst Rev.

[6] Sehat K, Baker RP, Pattison G, Price R, Harries WJ, Chesser TJS (2005). The use of the long gamma nail in proximal femoral fractures. Injury.

[7] Simpson AHRW, Varty K, Dodd CAF (1989). Sliding hip screws: modes of failure. Injury.

[8] Tencer AF, Johnson KD, Johnston DWC, Gill K (1984). A biomechanical comparison of various methods of stabilization of subtrochanteric fracture of the femur. J Orthop Res.

[9] Anglen JO, Weinstein JN (2008). Nail or plate fixation of intertrochanteric hip fractures: changing pattern of practice—a review of the American Board of Orthopedic Surgery Database. JBJS Am.

[10] Georgiannos Dimitrios, Lampridis Vasilios, Bisbinas Ilias (2014). Complications following Treatment of Trochanteric Fractures with the Gamma3 Nail: Is the Latest Version of Gamma Nail Superior to Its Predecessor?. Surgery Research and Practice.

[11] Pascarella R, Fantasia R, Maresca A, Bettuzzi C, Amendola L, Violini S (2016). How evolution of the nailing system improves results and reduces orthopedic complications: more than 2000 cases of trochanteric fractures treated with the Gamma Nail System. Musculoskelet Surg..

[12] Cipollaro L, Aicale R, Maccauro G, Maffulli N (2019). Single-versus double-integrated screws in intramedullary nailing systems for surgical management of extracapsular hip fractures in the elderly: a systematic review. J Biol Regul Homeost Agents.

[13] Aicale R, Maffulli N (2018). Greater rate of cephalic screw mobilization following proximal femoral nailing in hip fractures with a tip-apex distance (TAD) and a calcar referenced TAD greater than 25 mm. J Orthop Surg Res.

[14] Uzer G, Elmadag NM, Yıldız F, Bilsel K, Erden T, Toprak H (2015). Comparison of two types of proximal femoral hails in the treatment of intertrochanteric femur fractures. Ulus Travma Acil Cerrahi Derg.

[15] Buckley RE; Moran CG; Apivatthakakul T. AO Principles of fracture management. 3rd edition. Ed Thieme 2017. ISBN 3132423106, 9783132423107.

[16] Mereddy P, Kamath S, Ramakrishnan M, Malik H, Donnachie N (2009). The AO/ASIF proximal femoral nail antirotation (PFNA): a new design for the treatment of unstable proximal femoral fractures. Injury.

[17] Zhong B, Zhang Y, Zhang C, Luo CF (2014). A comparison of proximal femoral locking compression plates with dynamic hip screws in extracapsular femoral fractures. Orthop Traumatol Surg Res.

[18] Harris WH (1969). Traumatic arthritis of the hip after dislocation and acetabular fractures: treatment by mold arthroplasty. An end­result study using a new method of result evaluation. J Bone Joint Surg Am.

[19] Bellamy N. WOMAC Osteoarthritis index user guide. Version V. Brisbane, Australia 2002.

[20] Fogagnolo F, Kfuri M, Paccola CA (2004). Intramedullary fixation of pertrochanteric hip fractures with the short AO-ASIF proximal femoral nail. Arch Orthop Trauma Surg.

[21] Giessauf C, Glehr M, Bernhardt GA, Seibert FJ, Gruber K, Sadoghi P (2012). Quality of life after perthrocanteric femoral fractures treated with a gamma nail: a single center study of 62 patients. Muscol Disord.

[22] Buecking B, Bliemel C, Struwer J, Eschbach D, Ruchholtz S, Müller T (2012). Use of the Gamma3™ nail in a teaching hospital for trochanteric fractures: mechanical complications, functional outcomes, and quality of life. BMC Res Notes.

[23] Turgut A, Kalenderer O, Karapınar L, Kumbaracı M, Akkan HA, Aguş H (2016). Which factor is most important for occurrence of cutout complications in patients treated with proximal femoral nail antirotation? Retrospective analysis of 298 patients. Arch Orthop Trauma Surg.

[24] Ruecker AH, Rupprecht M, Gruber M, Gebauer M, Barvencik F, Briem D (2009). e treatment of intertrochanteric fractures: results using an intramedullary nail with integrated cephalocervical screws and linear compression. J Orthop Trauma.

[25] Megas P, Kaisidis A, Zouboulis P, Papas M, Panagopoulos A, Lambiris E (2005). Comparative study of the treatment of pertrochanteric fractures--trochanteric gamma nail vs. proximal femoral nail. Z Orthop Ihre Grenzgeb.

[26] Zhang S, Zhang K, Jia Y, Yu B, Feng W (2013). InterTan nail versus proximal femoral nail antirotation-Asia in the treatment of unstable trochanteric fractures. Orthopedics.

[27] Marsh JL, Slongo TF, Agel J, Broderick JS, Creevey W, DeCoster TA (2007). Fracture and dislocation classification compendium 2007: orthopedic trauma association classification, database and outcomes committee. J Orthop Trauma.

[28] Lindskog DM, Baumgaertner MR (2004). Unstable intertrochanteric hip fractures in the elderly. J Am Acad Orthop Surg.

[29] Kocher MS, Zurakowski D, Kasser JR (1999). Differentiating between septic arthritis and transient synovitis of the hip in children: an evidence-based clinical prediction algorithm. J Bone Joint Surg.

[30] Wu CC, Tai CL (2010). Effect of lag-screw positions on modes of fixation failure in elderly patients with unstable intertrochanteric fractures of the femur. J Orthop Surg.

[31] Nikoloski AN, Osbrough AL, Yates PJ (2013). Should the tip- apex distance (TAD) rule be modified for the proximal femoral nail antirotation (PFNA)?. A retrospective study. J Orthop Surg Res.

[32] Lorich DG, Geller DS, Nielson JH (2004). Osteoporotic pertrochanteric hip fractures: management and current controversies. Instr Course Lect.

